# Dietary Erucic Acid Induces Fat Accumulation, Hepatic Oxidative Damage, and Abnormal Lipid Metabolism in Nile Tilapia (*Oreochromis niloticus*)

**DOI:** 10.1155/2024/6670740

**Published:** 2024-06-19

**Authors:** Dingfei Ma, Qiangwei Li, Yuanyuan Xie, Youqin Kong, Zhili Ding, Jinyun Ye, Chenglong Wu, Yan Liu

**Affiliations:** National-Local Joint Engineering Laboratory of Aquatic Animal Genetic Breeding and Nutrition (Zhejiang) Huzhou University, 759 East 2nd Road, Huzhou 313000, China

## Abstract

Erucic acid (EA) in rapeseed oil has adverse effects on terrestrial animal and fish health. However, its antinutritional role in fish remains unclear due to the limited information on EA. Therefore, this study was conducted to assess the impact of EA on growth performance, antioxidative capacity, fatty acid profile, and lipid metabolism in tilapia. Six diets containing different amounts of EA (0, 3, 6, 12, 20, and 27 g/kg diet) were fed to tilapia (initial weight: 3.01 ± 0.01 g) for 8 weeks. The results exhibited that dietary EA did not affect growth performance but remarkedly increased the crude lipid contents (in the whole body, liver, and muscle). It also markedly increased the levels of low-density lipoprotein cholesterol, total cholesterol, nonesterified fatty acids, and triglyceride in the liver and serum in a dose-dependent manner. The EA groups had lower values of total superoxide dismutase, total antioxidant capacity, catalase, and higher activities of aspartate aminotransferase and alanine aminotransferase, as dietary EA levels increased. Feeding fish with diets containing EA (20 and 27 g/kg diet) significantly increased the malondialdehyde content. Moreover, dietary EA greatly altered the fatty acid profile in the liver and muscle. It especially elevated the percentages of C18 : 2n-6, C20 : 1n-9, and C22 : 1n-9 while decreasing the C18 : 0 and C16 : 0 levels. When the levels of EA in diets were 12, 20, and 27 g/kg, genes correlated with lipophagy, lipolysis, and *β*-oxidation were significantly reduced. Meanwhile, genes concerned in triglyceride synthesis were largely increased in the liver and muscle. In summary, high-dose EA (20 g/kg diet) in the diets significantly induced fat accumulation, hepatic oxidative damage, and abnormal lipid metabolism in tilapia. The current findings expand our understanding on the antinutritional role of EA in lipid homeostasis and fish health.

## 1. Introduction

Lipids serve as the sources of essential fatty acids and energy for development and health, as well as functional and structural molecules, macronutrients, and cellular messengers in aquatic animals [1]. Fish oil has been generally used as the primary lipid source, but due to its increasing price and decreasing supply, the proportion of fish oil is gradually reducing in the diets of farmed animals. Therefore, in recent decades, researches on replacing fish oil with plant oils have gained widespread attention. Plant oils (soybean oil and rapeseed oil) have advantages of high production and relatively low cost, making them the alternative lipid sources of fish oil in aquatic feed [2, 3, 4, 5]. Rapeseed oil abundant in unsaturated fatty acids including erucic acid (EA, 22 : 1n-9), oleic acid (C18 : 1n-9), and linoleic acid (C18 : 2n-6) is the third largest vegetable oil in the world, following soybean oil and palm oil [6]. There is increasing evidence that rapeseed oil has multiple health beneficial effects such as anticancer, neuroprotective, and antimicrobial in mammals, which is attributed to its great nutritional value [7, 8]. Furthermore, rapeseed oil is more readily available in larger amounts than soybean oil, because it is the most cultivated plant oil in China [9]. Therefore, the beneficial impacts and abundant production make rapeseed oil a better potential candidate for replacing fish oil in diets, after soybean oil.

In fact, a growing body of research on aquatic animals has indicated that rapeseed oil may cause adverse effects that could be detrimental to animal health [3, 4, 5, 10]. One of the most common negative effects observed in rapeseed oil-fed fish in the existing studies is excess lipid accumulation [3, 4]. For example, data demonstrate that the triglyceride content in serum was significantly increased in a research on the replacement of dietary fish oil by rapeseed oil in hybrid grouper [5]. Additionally, juvenile turbot and fingerling black carp fed diets containing rapeseed oil deposited more lipid in their liver than the fish oil group [4, 11]. In several studies on Atlantic salmon (*Salmo salar* L.), rapeseed oil in diets altered tissue fatty acid profile and the expression of genes concerned in hepatic lipid metabolism [10, 12, 13, 14]. Furthermore, feeding the large yellow croaker with rapeseed oil for 12 weeks decreased glutathion peroxidase, the total antioxidative capacity, and activities of superoxide dismutases 1 and 2, in the liver, which were due to fat deposition [3]. However, there is little information about the effects of rapeseed oil on fish, so it remains unknown that what leads to the aforementioned adverse impact induced by rapeseed oil-based diets.

It has been verified that the physiological effects of dietary oils are actually caused by their dominant fatty acids [2, 15]. For example, linoleic acid and palmitic acid were reported to be responsible for chronic inflammation and fat accumulation in fish fed with soybean oil and palm oil, respectively [16, 17]. Data from terrestrial animals reveal that rapeseed oil-induced adverse effects are closely associated with EA [18, 19]. EA is a monounsaturated omega-9 fatty acid (22 : 1n-9) mainly distributed in high EA-rapeseed oil (ranging from 20% to 54%) [20, 21]. Some studies suggest that EA diets cause a decreased oxidation capacity of mitochondria and fat accumulation in the heart [22, 23, 24]. The accumulated lipid impairs lipid metabolism, thus further inducing more formation of fat in a dose-dependent manner [25]. Furthermore, high intake of EA changes lipid contents and fatty acid composition, which has been confirmed in laying hens and rats [24, 26, 27, 28]. However, it has attracted less attention in studies of aquatic animals, due to the lack of precise reporting on the concentration of EA [3, 4, 5, 10, 12, 14]. Fortunately, one recent study in grass carp has verified that EA in diets is harmful to fish health, suppressing growth and initiating an inflammation response in the intestine [29]. Based on the above, we speculate fat deposition and altered lipid metabolism in rapeseed oil-fed fish might be attributed to high content of EA in rapeseed oil.

In the current study, Nile tilapia was used to verify our hypothesis that EA in rapeseed oil could result in fat deposition and altered lipid metabolism in fish. Additionally, soybean oil is widely used as the fat source in the artificial diet of tilapia [30, 31]. As the production of farmed tilapia increases, there is also an increasing demand for soybean oil. Therefore, it is necessary to identify other suitable vegetable oils to ensure the continued growth of tilapia production. For this reason, this study chose soybean oil and rapeseed oil as the fat sources for Nile tilapia. After 8-week culture experiment, we carried out a systematic analysis of growth performance, biochemical parameters, fatty acid profile, and the expressions of genes involved in lipid metabolism. The results of this study will be conducive to a better understanding of the antinutritional role of dietary EA in aquatic animals and provide valuable information for improving fish health and the utilization of rapeseed oil-based diets rich in EA.

## 2. Materials and Methods

### 2.1. Diets and Preparation Procedure

Experiment diets were made at the National-Local Joint Engineering Laboratory of Aquatic Animal Genetic Breeding and Nutrition (Huzhou, China). The feed formula and its fatty acid profile in our study are presented in Tables 1 and 2, respectively. Low EA rapeseed oil and soybean oil were selected as the basic dietary oils. According to the EA contents tested (ranging from 6 to 400 g/kg) in rapeseed oils sold in the Chinese market, various concentrations of EA were put to the basic oils of the experimental diets. EA was purchased from Aladdin Reagent Co., Ltd., in China (80% purity), and Thermo Fisher, in the USA (90% purity), and the two sources were mixed in a 1 : 1 ratio. Palmitic acid was also added to maintain equal levels of dietary lipids in all diets. The actual values detected of EA (g/kg diet) in diets are 0 (EA0), 3 (EA1), 6 (EA2), 12 (EA3), 20 (EA4), and 27 (EA5), respectively.

### 2.2. Culture Experiment

The experimental procedures were conducted according to the guidelines for the Care and Usage of Laboratory Animals in China and approved by the Animals Ethics Committee of Huzhou University. The juvenile tilapias were purchased from Freshwater Fisheries Research Center of the Chinese Academy of Fishery Sciences in Jiangsu Province, China. Fish underwent 2 weeks of domestication in the circulating water system and were fed by a tilapia diet (Tongwei. Co. Ltd., Chengdu, China). After 2 weeks, 540 fish (3.01 ± 0.01 g) were randomly allocated to 18 tanks, in triplicates per treatment (30 fish per tank). Tilapias were cultured for 8 weeks using our feed containing different amounts of EA.

### 2.3. Sample Collection

After 8 weeks, 16 fish were randomly chosen from each experimental group and anesthetized with diluted tricaine methanesulfonate (MS-222, Sigma, USA) and weighed for calculating parameters of growth performance. Blood centrifugation condition was 1,000 *g* for 10 min. Liver and muscle samples were taken for histological observation, chemical analysis, fat level determination, fatty acid content analysis, and mRNA level studies.

### 2.4. Measurement of Serum and Liver Biochemical Indicators

Biochemical indeces including triglycerides (TG), total cholesterol (TC), low-density lipoprotein cholesterol (LDL-C), high-density lipoprotein cholesterol (HDL-C), glucose (GLU), nonesterified fatty acid (NEFA), malondialdehyde (MDA), alanine aminotransferase (ALT), aspartate aminotransferase (AST), total antioxidant capacity (T-AOC), total superoxide dismutase (T-SOD), catalase (CAT), and hydrogen peroxide (H_2_O_2_) in the serum and liver of tilapia were detected using commercial assay kits provided by Jiancheng Bioengineering Institute (Nanjing, China). Furthermore, the serum insulin (INS) content was measured using an enzyme-linked immunosorbent assay (ELISA) (Shanghai Hengyuan Biotechnology Co. Ltd., Shanghai, China).

### 2.5. Proximate Composition and Fatty Acid Profile

The crude protein, moisture, and ash levels of the whole body and tissues in fish and diets were detected as follows. After processing the samples of fish to the appropriate size, the samples were weighed (W1) and then freeze-dried using a vacuum freeze dryer under −80°C and 0.1 kPa conditions (Marin Christ Alpha2-4 LSC Basic, Germany). They were weighed every 3 days until they reached a constant weight (W2). The moisture content could be obtained according to the weight difference between W1 and W2. The ash contents were measured by the muffle furnace firing method at 550°C. The samples after vacuum freeze-drying were carbonized completely on an electric furnace and then placed in a muffle furnace (Neytech Vulcan Benchtop Furnace Model 3-1750 #9493409, USA) at 550°C for 6 hr. The dried samples were ground into a uniform powder, and their crude protein contents were assessed by analyzing the total nitrogen (N) content using the Dumas method (Elementar Rapid N exceed, Germany), followed by the calculation of the percentage crude protein. Extraction of the lipids from the experimental diets, whole body, muscles, and livers with a chloroform/methanol (2/1, v/v) mixture was conducted as previously presented [2]. The fatty acid methyl esters (FAMEs) were then obtained by derivatization with 14% boron trifluoride-methanol, according to our previous work [16]. Detection of the FAMEs was finished on gas chromatography (Agilent 8890, USA). The chromatographic column and other detection parameters were described previously [16]. The area percentage of FAMEs indicated the relative content of individual fatty acid.

### 2.6. Liver Histological Analysis

Livers were sampled (1 cm) (three fish each group) and immediately put in 4% paraformaldehyde for 24 hr. After fixation, the samples were sent to Hangzhou Haoke Biotechnology Co., Ltd. for being stained with hematoxylin and eosin (H&E). To measure the relative areas of lipid droplets in the sections, ImageJ software was utilized.

### 2.7. Analysis of mRNA Levels

The real-time quantitative RT-PCR assay in tissues was finished according to a previous study [2]. The mRNA levels of genes associated with lipid catabolism and lipogenic capacity were assessed. This study chose the house-keeping gene elongation factor 1 alpha (ef1*α*) and *β*-actin as the reference genes.  Table 3 summarizes the primer information of the genes used in our study. The relative levels of the targeted genes were obtained with the 2^–*ΔΔ*Ct^ method as previously described [32].

### 2.8. Statistical Approach

Statistical analysis of all data was conducted using a one-way analysis of variance (ANOVA). When significant differences were shown, the Tukey's multiple range test was chosen to compare dietary treatments (SPSS software package, version 26.0).

## 3. Results

### 3.1. Growth Performance

As the level of EA in the diet increased from the EA0 group to the EA4 group, we observed a tendency of elevated mesenteric fat index (MFI) and hepatosomatic index (HSI). Among them, the EA4 treatment group showed remarkedly higher values than the EA0 group (Table 4). However, no significant differences were observed in weight gain (WG), specific growth rate (SGR), and feed efficiency (FE) among all groups.

### 3.2. Proximate Composition and Histological Observation

**Figure 1:**
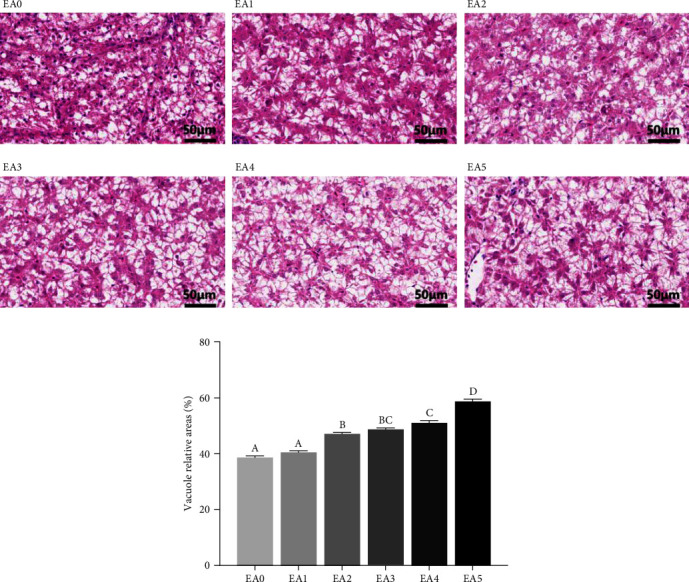
Effects of dietary EA levels on lipid content in the liver of tilapia. (a) The liver slices were treated with hematoxylin and eosin (H&E) staining. Scale bars, 50 *μ*m. (b) The relative area of H&E staining lipid droplets. After H&E staining, the nucleus was blue. EA0, EA1, EA2, EA3, EA4, and EA5: the experimental groups with different contents of EA (0, 3, 6, 12, 20, and 27 g/kg diet). Values are presented as means ± SEM (*n* = 6), and different capital letters above the bars (*P* < 0.05).

Effects of dietary EA on proximate compositions of the whole body, muscle, and liver in tilapia are summarized in Table 5. The EA groups exhibited lower whole-body moisture contents, and the EA5 group achieved the lower value than the EA1 group. When compared to the EA0 group, the whole body and muscle protein levels in all EA groups (except whole body protein in the EA2 group) were dramatically decreased, resulting in fish fed the EA diets having lower protein content. Conversely, as the dietary EA level increased, the lipid contents of the whole body, muscle, and liver in fish fed the EA diets increased significantly. The EA5 group showed the higher values for the lipid level in both the whole body and muscle than the EA0 and EA1 groups and had the highest liver lipid content compared to the other groups. Histological analysis of the liver indicated that dietary EA (especially at levels EA3, EA4, and EA5) clearly increased lipid droplet area compared to the control group (EA0) (Figure 1), aligning with the result for liver lipid content presented in Table 5.

### 3.3. Biochemical Indicators

The dietary EA levels markedly affected the contents of TG, TC, LDL-C, HDL-C, NEFA, GLU, H_2_O_2_, MDA, and insulin as well as the activities of T-AOC, T-SOD, CAT, AST, and ALT in the serum and liver of tilapia (Table 6). With increasing dietary EA levels, the TG, TC, LDL-C, and insulin values in the serum of fish were elevated significantly, with the EA5 group having the highest values. Similar tendencies were observed in the values of NEFA, MDA, and H_2_O_2_ in the serum. Feeding fish with diets containing EA significantly reduced the HDL-C and glucose contents in the serum, and the EA5 group (27 g/kg diet) exhibited the lowest values among all groups. The activities of T-AOC, T-SOD, and CAT in serum of fish were reduced significantly with increased EA levels in the diet. All EA groups showed lower activity of T-SOD than the EA0 group. Lower activities of T-SOD and CAT both were found in the EA4 group (20 g/kg diet). In contrast to the antioxidant enzyme activities, dietary EA significantly elevated the activities of AST and ALT (markers of liver injury) in the serum. Dietary EA also greatly altered lipid contents and antioxidant enzyme activities in the liver of fish. The tendencies of TG, TC, NEFA, MDA, and H_2_O_2_ contents and T-AOC, T-SOD, and CAT activities in the liver of fish were similar to those observed in serum.

### 3.4. Fatty Acid Profile

The composition of fatty acid in the liver and muscle of fish was dramatically changed by dietary EA, as shown in Tables 7 and 8, respectively. Notably, the majority of fatty acids differed significantly among the six experimental groups. In EA5 diet-fed fish, the liver had lower percentages of 16 : 0, 18 : 0, and 18 : 1n-9 and higher percentages of 14 : 0, 18 : 2n-6, 18 : 3n-3, 18 : 3n-6, 20 : 1n-9, 20 : 3n-6, 20 : 5n-3, and 22 : 1n-9 (Table 7). Consequently, the total levels of saturated fatty acids (SFA) and monounsaturated fatty acids (MUFA), polyunsaturated fatty acids (PUFA), n-3 PUFA, and n-6 PUFA were lower and higher in the liver of EA5-fed fish, respectively. The profile of fatty acid in muscle was similar to that in the liver, except that the 18 : 1n-9 content was increased (Table 8).

### 3.5. Effect of Dietary EA on Lipid Metabolism in the Liver and Muscle

**Figure 2:**
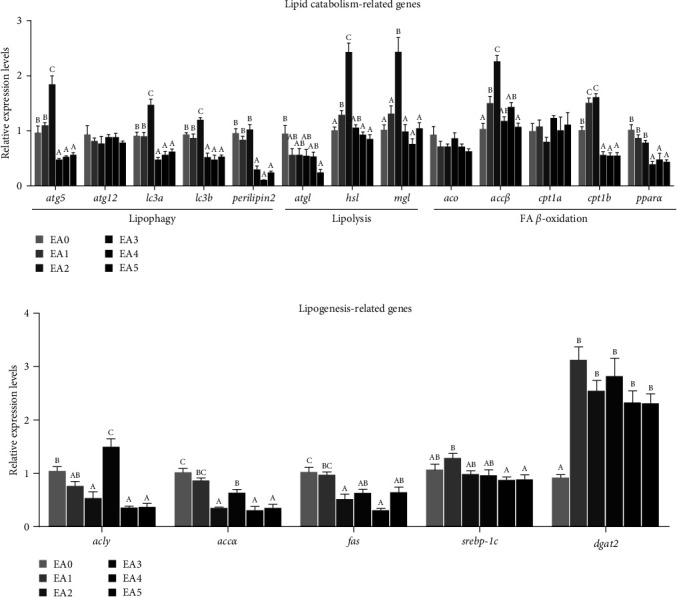
Effects of dietary EA levels on the expressions of genes related to lipid metabolism in the liver of tilapia. (a) Lipid catabolism-related genes. (b) Lipogenesis-related genes. EA0, EA1, EA2, EA3, EA4, and EA5: the experimental groups with different contents of EA (0, 3, 6, 12, 20, and 27 g/kg diet). *atg*5, autophagy-related 5; *atg*12, autophagy-related 12; *lc*3*a*, microtubule-associated protein 1 light chain 3 alpha; *lc*3*b*, microtubule-associated protein 1 light chain 3 beta; *perilipin*2; *atgl*, adipose triglyceride lipase; *hsl*, hormone-sensitive lipase; *mgl*, monoacylglycerol lipase; *aco*, acyl-CoA oxidase; *accβ*, acetyl-CoA carboxylase beta; *cpt*1*a*, carnitine palmitoyltransferase 1a; *cpt*1*b*, carnitine palmitoyltransferase 1b; *pparα*, peroxisome proliferator-activated receptor *α*; *acly*, ATP citrate lyase; *accα*, acetyl-CoA carboxylase *α*; *fas*, fatty acid synthase; *dgat*2, diacylglycerol O-acyltransferase 2; *srebp*-1*c*, sterol regulatory element-binding transcription factor 1c. Values are presented as means ± SEM (*n* = 8), and different capital letters above the bars (*P* < 0.05).

**Figure 3:**
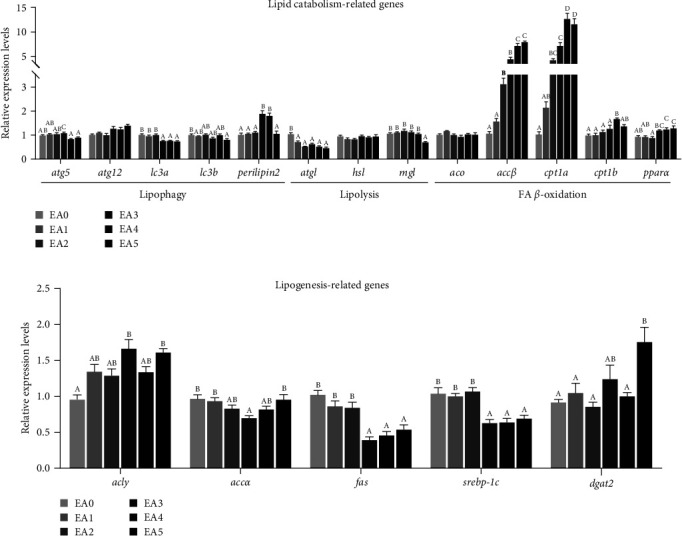
Effects of dietary EA levels on the expressions of genes related to lipid metabolism in the muscle of tilapia. (a) Lipid catabolism-related genes. (b) Lipogenesis-related genes. EA0, EA1, EA2, EA3, EA4, and EA5: the experimental groups with different contents of EA (0, 3, 6, 12, 20, and 27 g/kg diet). *atg*5, autophagy-related 5; *atg*12, autophagy-related 12; *lc*3*a*, microtubule-associated protein 1 light chain 3 alpha; *lc*3*b*, microtubule associated protein 1 light chain 3 beta; perilipin2; *atgl*, adipose triglyceride lipase; *hsl*, hormone-sensitive lipase; *mgl*, monoacylglycerol lipase; *aco*, acyl-CoA oxidase; *accβ*, acetyl-CoA carboxylase beta; *cpt*1*a*, carnitine palmitoyltransferase 1a; *cpt*1*b*, carnitine palmitoyltransferase 1b; *pparα*, peroxisome proliferator-activated receptor *α*; *acly*, ATP citrate lyase; *accα*, acetyl-CoA carboxylase *α*; *fas*, fatty acid synthase; *dgat*2, diacylglycerol O-acyltransferase 2; *srebp*-1*c*, sterol regulatory element-binding transcription factor 1c.Values are presented as means ± SEM (*n* = 8), and different capital letters above the bars (*P* < 0.05).

The gene expression of lipid metabolism in the liver and muscle was influenced by dietary EA, as illustrated in Figures 2 and 3. The genes involved in lipophagy (autophagy protein 5 (*atg*5), microtubule-associated protein 1 light chain 3 alpha (*lc*3*a*), microtubule-associated protein 1 light chain 3 beta (*lc*3*b*), and *perilipin*2), lipolysis (hormone-sensitive lipase (*hsl*) and monoglyceride lipase (*mgl*)), and *β*-oxidation (acetyl-CoA carboxylase beta (*accβ*) and carnitine palmitoyl transferase I beta (*cpt*1*b*)) were significantly up- and downregulated, respectively, with increasing EA contents in the diet from 0 (EA0) to 6 g/kg (EA2) and from 12 (EA3) to 27 g/kg (EA5) in the liver (Figure 2(a)). The mRNA levels of adipose triglyceride lipase (*atgl*) and peroxisome proliferator-activated receptor alpha (*pparα*) were downregulated as the dietary EA content increased. There was a contrasting trend in the expression of genes related to lipogenesis (ATP citrate lyase (*acly*), acetyl-CoA carboxylase alpha (*accα*), fatty acid synthase (*fas*), and sterol regulatory element-binding transcription factor 1 c (*srebp*-1*c*)), which were greatly reduced in the liver (Figure 2(b)). However, compared to the EA0 group, all EA groups showed higher mRNA levels of diacylglycerol O-acyltransferase 2 (*dgat*2) in the liver. The dietary high-dose EA (12 g/kg diet) increased the *accβ*, *cpt*1*a*, *cpt*1*b*, *pparα*, *perilipin*2, and *acly* mRNA levels and decreased the *lc*3*a*, *lc*3*b*, *atgl*, *accα*, *fas*, and *srebp*-1*c* mRNA levels in the muscle (Figure 3).

## 4. Discussion

### 4.1. Effects of EA on Growth Performance and Lipid Accumulation in Tilapia

The influence of EA on growth performance has been assessed in animals, and most results indicate that dietary EA can reduce weight gain [19, 29, 33]. Chicks and rats fed diets containing high levels of EA exhibited slower growth rates and decreased body weight gain [19, 33]. Nevertheless, one study found that EA increased body weight gain in rats [25]. More recently, data from fish experiments have shown that diets containing more than 6 g/kg of EA significantly inhibited growth in grass carp, and the degree of suppression was positively correlated with the EA level in the diet [29]. In contrast, our study found that dietary EA did not change the growth of tilapia, even when its contents in the diet were up to 27 g/kg, which is inconsistent with previous studies. The inconsistent results regarding the effect of EA on weight gain in available studies indicate that there are species-specific differences in the utilization of EA. Our data suggest that tilapia is not sensitive to high levels of EA in terms of growth performance, which may have implications for the potential use of rapeseed oil containing high erucic acid.

The majority of studies have demonstrated that a high intake of EA is closely relevant to more fat deposits in tissues in animals [25, 27, 28, 34]. However, the effect of dietary EA on fat accumulation in fish has received little attention. In this study, we found that EA supplementation in diets induced lipid deposition in fish, with a significantly higher value observed in the high-level group, as previous studies reported [26, 29]. Above all, HSI, MFI, and the lipid levels of whole fish, liver, and muscle were positively correlated with dietary EA levels. Secondly, the histological evaluation of hepatic H&E staining exhibited that dietary EA significantly increased fat accumulation by elevating the number and size of hepatic lipid droplets. Furthermore, the EA-treated fish had marked increases in TG, TC, LDL-C, and NEFA contents of the serum and liver. These findings suggested that high levels of EA also inhibited lipid utilization and induced fat deposition in fish. This might be the reason why rapeseed oil has adverse effects on some fish [3, 4, 5], which is most probably attributed to its high EA content. In general, impaired lipid utilization can increase glucose utilization as a compensatory mechanism to maintain energy homeostasis, as documented in previous studies [30, 31, 35]. Accordingly, this study suggests that the EA treatment increased the insulin level and decreased the glucose concentration in serum, indicating that lipid-sourced energy was decreased. Additionally, we found that the protein levels of the whole body and muscle in the EA-treated groups were also compensatorily decreased in response to suppressed lipid utilization. A study performed on chicks also revealed that the high EA treatment deposited significantly less carcass protein [18], in accordance with our findings. The results indicated that a high level of dietary EA led to increased fat deposition and a decrease in glucose and protein concentrations. In short, this study suggested that high and prolonged intake of EA in tilapia did not affect growth performance, but it did greatly promote excess fat accumulation.

### 4.2. EA Decreases Antioxidant Capacity in Tilapia

The antioxidant status was also used to test the effect of dietary EA on animal health, in addition to its effect on growth performance and lipid accumulation. Increasing evidence indicates that the accumulation of fat caused by dietary EA can diminish antioxidant capacity and significantly reduce the activity of antioxidant enzyme [3, 5, 26, 29]. In this study, we examined biochemical indicators of serum and liver to study the impact of dietary EA on the antioxidant status of fish. Our findings indicated that EA elevated the levels of MDA, a lipid peroxidation product, in both the serum and liver. Furthermore, the activities of three key enzymes, T-AOC, SOD, and CAT, in the antioxidative enzyme system were all markedly reduced in the serum and liver of EA-treated fish. A previous study on grass carp also reported that EA elevated the MDA contents accompanied by lower antioxidant capacity with the reduced activities of SOD and CAT, in line with our findings [29]. Conversely, hydrogen peroxide—a byproduct of oxidase enzymes—was observed to be particularly elevated in the high-dose EA groups (EA4 and EA5) of the present study. High levels of hydrogen peroxide formation in EA-treated fish suggested a potential lack of CAT, the primary enzyme responsible for breakdown of hydrogen peroxide into water [36]. In fact, our data revealed that hydrogen peroxide stress caused by decreased CAT activity may induce hydrogen peroxide toxicity. It has been reported that overabundant hydrogen peroxide can directly oxidize unincorporated intracellular ferrous iron, potentially leading to oxidative stress [36]. Therefore, lower antioxidant activity and hydrogen peroxide stress may intensify oxidative stress in the high-dose EA groups. Moreover, this study is the first to demonstrate that long-term intake of EA can elevate ALT and AST activities in fish, indicating the presence of hepatic oxidative injury. Previous studies have established a link between hepatic oxidative damage and lipid peroxidation resulting from excessive lipid accumulation [15, 31, 37, 38]. Consequently, we postulate that the abnormal lipid deposition caused by dietary EA promotes lipid peroxidation and oxidative stress, which subsequently impairs antioxidant systems and ultimately induces hepatic damage. This mechanism is supported by previous studies [39, 40, 41]. However, the precise underlying mechanism remains elusive and requires further exploration. In short, our data suggest that a high level of EA in diets may increase the risk of oxidative damage in fish.

### 4.3. EA Changes Fatty Acid Composition in Tissues

There are several earlier researches which have investigated the impact of EA on fatty acid composition in chicks and rats [19, 24, 27, 33, 42, 43], but until now, there has been no research on its effects in fish. In this study, we found that dietary EA not only affected lipid accumulation in tissues but also largely changed the fatty acid content in liver and muscle of fish. Previous studies in rats have clearly shown that EA (C22 : 1n-9) can be chain shortened into C20 : 1n-9 (eicosenoic acid), C18 : 1n-9, and C18 : 0 (stearic acid) [42, 43]. This is consistent with our findings, as we observed significant increases in EA (in the muscle and liver), C20 : 1n-9 (in muscle and liver), and C18 : 1n-9 (in muscle) in fish treated with EA. Notably, the EA treatment markedly decreased the C18 : 1n-9 level in the liver of fish, which may be due to the fish preferentially oxidizing this fatty acid in response to impaired lipid utilization, as our previous study suggested [31]. In the present study, there might be preferential utilization of C18 : 1n-9 from diets or EA chain shortening as an energy source in the liver of fish. This contributed to lower C18 : 1n-9 when dietary EA suppressed lipid utilization. The C18 : 0 content (another chain-shortened metabolite of EA) was reduced in the liver and muscle of EA-treated fish. Data from chicks fed with rapeseed oil containing 22.3% EA were consistent with our findings, where decreased C18 : 0 was observed [27]. This suggested that dietary EA also accelerated the utilization of C18 : 0 for energy provision, thus reducing its deposition in tissues. Additionally, experiments performed on neural cells of humans and chicks both revealed that the chain of EA could be elongated to C24 : 1n-9 (nervonic acid) [44, 45]. In fish, less or no EA chose to undergo elongation metabolism, so there was no C24 : 1n-9 detected in tissues of fish in our study. Our findings demonstrated, for the first time, that the concentrations of EA, C20 : 1n-9, and C18 : 0 in tissues of fish were positively and negatively correlated with dietary EA levels, respectively, in accordance with a previous study [27]. This study also found that feeding EA diets led to higher contents of C14 : 0, C18 : 2n-6, C18 : 3n-3, C18 : 3n-6, and C20 : 5n-3 and lower levels of C16 : 0. It could be explained that in the EA-treated groups, C16 : 0 might be preferentially oxidized for energy provision, and more other fatty acids (including C14 : 0, C18 : 2n-6, C18 : 3n-3, C18 : 3n-6, and C20 : 5n-3) would be deposited. In conclusion, EA dramatically altered the fatty acid contents in tissues of fish.

### 4.4. EA Induces Abnormal Lipid Metabolism in Fish

This study and previous animal studies have proved that dietary EA contributes to lipid deposition [25, 27, 28, 34]. It has been demonstrated that excess fat accumulation is closely linked to abnormal lipid metabolism [2, 25, 31, 35, 37]. Yet, there are few reports studying the impact of dietary EA on lipid metabolism in fish. The objective of this current study was to investigate the expressions of the genes relevant to lipid catabolism and lipogenesis in the liver and muscle. This is the first study to systematically investigate the role of EA in lipid metabolism in fish.

It has been reported that EA, as a very long-chain fatty acid, is preferentially oxidized in peroxisomes, which can increase peroxisomal *β*-oxidation [46, 47]. Some studies in early have demonstrated that feeding animals diets rich in EA enhances the activity of peroxisomal *β*-oxidation [47, 48]. In this study, the expression of *accβ* (the rate-limiting enzyme of peroxisomal *β*-oxidation) was dramatically elevated in the liver of the low-dose EA groups (EA1 and EA2). This indicated that low level of EA in the diet increased peroxisomal *β*-oxidation of the liver of fish, in accordance with previous studies [46, 47]. However, the mRNA levels of *accβ* were remarkably decreased in the liver of the high-dose groups (EA3, EA4, and EA5). This may be because peroxisomal *β*-oxidation was overactivated by the high levels of EA, leading to a significant increase in peroxisome-derived H_2_O_2_ content [37] (Table 6) and potentially increasing the risk of oxidative damage [15, 49]. To prevent excessive oxidative stress derived from peroxisomes, the reduced expression of *accβ* can be considered as a feedback mechanism. The lower mRNA level of *pparα* (the transcriptional regulation factor of peroxisomal *β*-oxidation) also indicated that EA at high concentrations (EA3, EA4, and EA5) inhibited peroxisomal *β*-oxidation function in the liver. Conversely, peroxisomal fatty acid oxidation was widely enhanced in mammals fed a high-EA rapeseed oil diet, where *pparα* was activated [50, 51]. The difference can be attributed to different EA contents and species tolerance to EA. Studies suggest that peroxisomal oxidation of EA inhibits fatty acid oxidation in the mitochondria, liver, and heart by stimulating malonyl-CoA formation [22, 23, 25, 33, 52]. In this study, we found that dietary high-dose EA caused significantly downregulation of *cpt*1*b* (the rate-limiting enzyme of mitochondrial *β*-oxidation) in the liver of the EA3, EA4, and EA5 groups, suggesting that mitochondrial *β*-oxidation was decreased. This is the reason why the high-dose EA groups had more NEFA deposited in the serum and liver (Table 6). On contrary to the liver, the upregulation of *cpt*1*a* and *cpt*1*b* was observed accompanied by higher *accβ* and *pparα* expressions in the muscle of the EA-fed fish, implying that peroxisomal and mitochondrial *β*-oxidation were enhanced. However, the higher mRNA expression did not contribute to increased *β*-oxidation activities in peroxisomes and mitochondrion, because the higher lipid contents were found in the muscle (Tables 5 and 8). Additionally, as a previous study reported in rats [43], the liver might also be the predominant organ for metabolizing EA in this study. Therefore, more EA was preferred to be oxidized in the liver (Table 7), which probably resulted in different impacts of EA on the expression patterns of genes concerned in fatty acid *β*-oxidation.

Our study also found that the expressions of key enzymes in lipophagy (*atg*5, *lc*3*a*, *lc*3*b*, and *perilipin*2) and lipolysis (*hsl* and *mgl*) were largely affected by EA in the liver and muscle. These enzymes play crucial roles in lipid catabolism and have an impact on lipid contents [53, 54]. The expression trends of these genes are similar to those of *accβ* and *cpt*1*b* in the liver of EA-treated fish. The downregulation of genes associated with lipophagy and lipolysis further contributed to lipid accumulation in the tissues of fish fed with high levels of EA. Correspondingly, we observed that the high-dose EA groups had higher lipid deposits in the liver and muscle (Tables 7 and 8). Lipid metabolism is also involved in fatty acid or lipid production, and its dysfunction can lead to increased lipid levels (including NEFA and TG) [25, 31, 35]. Previous researches have mainly paid attention on the effect of EA on lipid catabolism [25, 27, 52], but the effect of EA in diets on lipogenesis in animals remains unclear. In our study, we found that *srebp*-1*c* and its targeted lipogenic genes (*acly*, *accα*, and *fas*) were markedly decreased in the liver and muscle of fish fed with the EA diets. This suggests that fatty acid synthesis was inhibited in response to increased NEFA in the serum and liver (Table 6). *dgat*2 is closely associated with the accumulation of TG, as it is a key enzymes for TG synthesis [55]. Our study exhibited that dietary EA enhanced *dgat*2 expression in the liver and muscle. This finding provides an explanation for why EA-treated fish have higher TG or total lipids in serum and tissues. In summary, our results demonstrate that dietary high-dose EA promotes lipid deposition by increasing TG synthesis and reducing lipophagy, lipolysis, and *β*-oxidation.

## 5. Conclusion

In conclusion, our results suggest that high-dose EA (20 g/kg diet) in diets promotes fat accumulation, reduces antioxidant capacity, causes hepatic damage, and disrupts normal lipid metabolism in tilapia. These findings enhance our understanding of the antinutritional role of EA in fish health and lipid homeostasis. They also provide valuable information for nutritional therapies aimed at improving the utilization of high erucic acid rapeseed oil in farmed fish.

## Figures and Tables

**Table 1:** Formulation and proximate analysis of the experimental diets.

Ingredients (g/kg)	EA0	EA1	EA2	EA3	EA4	EA5
Casein	364	364	364	364	364	364
Gelatin	91	91	91	91	91	91
Corn starch	320	320	320	320	320	320
Soybean oil	20	20	20	20	20	20
Rapeseed oil	20	20	20	20	20	20
Lecithin high potency	5	5	5	5	5	5
Butylated hydroxytoluene	0.2	0.2	0.2	0.2	0.2	0.2
Mineral premix^a^	15	15	15	15	15	15
Vitamin premix^b^	20	20	20	20	20	20
Carboxy methyl cellulose	30	30	30	30	30	30
Choline chloride	5	5	5	5	5	5
Calcium dihydrogen phosphate	15	15	15	15	15	15
Cellulose	54.8	54.8	54.8	54.8	54.8	54.8
Erucic acid^c^	0	4.7	9.4	18.8	28.2	35
Palmitic acid	35	30.3	25.6	16.2	6.8	0
Attractant	5	5	5	5	5	5
Total	1,000	1,000	1,000	1,000	1,000	1,000
Proximate analysis						
Crude protein (g/kg)	390.8	388.1	393.0	398.1	391.8	391.7
Crude lipid (g/kg)	80.2	79.7	80.9	80.7	79.2	80.3
Moisture (g/kg)	70.3	70.0	69.2	68.7	68.3	69.3
Ash (g/kg)	39.1	40.0	40.2	40.7	40.0	39.8

*Note*. ^a^Mineral premix (g/kg): KCl 28 g, MgSO_4_ · ·7H_2_O 100 g, NaH_2_PO_4_ 215 g, KH_2_PO_4_ 100 g, Ca (H_2_PO_4_) _2_ · H_2_O 265 g, CaCO_3_ 105 g, C_6_H_10_CaO_6_ · ·5H_2_O 165 g, FeC_6_H_5_O_7_ · ·5H_2_O 12 g, ZnSO_4_ · ·7H_2_O 4.76 g, MnSO_4_ · H_2_O 1.07 g, AlCl_3_ · ·6H_2_O 0.15 g, CuCl_2_ · ·2H_2_O 2.4 g, CoCl_2_ · ·6H_2_O 1.4 g, KI 0.23 g, *α*- cellulose 0.043 g. ^b^Vitamin premix (g/kg): VA 1.29 g, VC 57.5 g, VE 20 g, VD_3_ 0.63 g, VK_3_ 1.8 g, VB_1_ 7.5 g, VB_2_ 2.63 g, VB_6_ 1 g, VB_12_ 0.15 g, nicotinic acid 5 g, folic acid 0.19 g, inositol 60 g, biotin 0.75 g, calcium pantothenate 5 g, p-aminobenzoic acid 5 g, *α*-cellulose 831.56 g. ^c^ Erucic acid was purchased Aladdin Reagent Co., Ltd., in China (80% purity), and Thermo Fisher, in the USA (90% purity), and the two sources were mixed in a 1 : 1 ratio. EA0, EA1, EA2, EA3, EA4, and EA5: the experimental groups with different contents of EA (0, 3, 6, 12, 20, and 27 g/kg diet).

**Table 2:** Fatty acid composition (total fatty acids (g (kg))) of six experimental diets with different erucic acids.

Fatty acids	EA0	EA1	EA2	EA3	EA4	EA5
C12 : 0	3.2	3.1	3.0	3.2	3.3	3.6
C14 : 0	12.2	11.5	11.4	11.6	11.6	12.0
C16 : 0	548.6	502.1	452.6	337.0	196.9	95.6
C16 : 1n-7	3.4	3.1	3.2	3.4	3.5	3.8
C18 : 0	27.1	26.2	26.4	27.8	28.7	29.8
C18 : 1n-9	186.9	191.0	196.1	210.3	226.3	236.9
C18 : 2n-6	177.8	185.3	190.1	204.8	219.5	226.3
C18 : 3n-3	34.0	35.9	36.9	40.0	43.1	45.1
C20 : 0	2.3	2.5	2.4	2.7	3.3	3.7
C20 : 1n-9	4.8	5.3	5.9	7.5	9.2	10.4
C22 : 1n-9	0	34.4	72.4	152.0	254.6	333.2
Total SFA	593.3	545.3	495.7	382.2	243.8	144.6
Total MUFA	195.0	233.7	277.4	373.1	493.6	584.3
Total PUFA	211.8	221.1	227.0	244.8	262.6	271.4
Total n-3 PUFA	34.0	35.9	36.9	40.0	43.1	45.1
Total n-6PUFA	177.8	185.3	190.1	204.8	219.5	226.3
n-3/n-6	0.19	0.19	0.19	0.20	0.20	0.20

*Note*. MUFA, monounsaturated fatty acids; PUFA, polyunsaturated fatty acids; SFA, saturated fatty acids. Total SFA includes 12 : 0, 14 : 0, 16 : 0, 18 : 0, and 20 : 0; total MUFA includes 16 : 1n-7, 18 : 1n-9, 20 : 1n-9, and 22 : 1n-9; total PUFA includes 18 : 2n-6, and 18 : 3n-3; total n-3 PUFA includes 18 : 3n-3; total n-6 PUFA includes 18 : 2n-6. EA0, EA1, EA2, EA3, EA4, and EA5: the experimental groups with different contents of EA (0, 3, 6, 12, 20 and 27 g/kg diet).

**Table 3:** Primers used for the analysis of mRNA gene expression by qRT-PCR.

Gene name	Forward primer	Reverse primer	Size (bp)	GenBank no.
*atg*5	ACAGCGTCTTACCCTGGAGCA	TCACAGAGCTGGATGGGCAGT	84	XM_003450274.5
*atg*12	TCATATCTCGCTTCCTCAAGC	CCCTACTTCTTGACTCGGTGA	92	XM_025911479.1
*lc*3*a*	GTCCAGCAGATCCGTGAGC	TGCCAGGAACTTGGTCTTGTC	105	XM_019356796.2
*lc*3*b*	GCCTCCAGCTAAACTCCAACC	CGCTCTCGCTCGTACACCTC	100	XM_003439438.5
*perilipin*2	AGCCTCAGATTGCTATGGCCAAT	TGGTAGAGAATCGGCAGGGTT	95	KF751704
*atgl*	AAAACGTCCTGGTGACCCAGT	TAGGAGGAATGATGCCACAGTACA	104	XM_003440346
*hsl*	AACCTGGATGTCCATTTCTGGAAG	TCGGTTTACCTTGACTTGAGTGGA	102	FJ601660
*mgl*	ACATCGTCAACGCAGACGGATT	CACAATGTTCCCCAGCTCCAT	105	XM_005478351.1
*aco*	AGTCCCACTGTGAGCTCCATCAA	CAGACCATGGCAGTTTCCAAGA	108	KF918710
*accβ*	ACATGCAGTCCATGCTGCGT	AAATGCCTCTCAAGCCACTCAA	106	XM_003451659
*cpt*1*a*	TTTCCAGGCCTCCTTACCCA	TTGTACTGCTCATTGTCCAGCAGA	102	XM_003440552
*cpt*1*b*	AAGGGACGTTACTTCAAGGTG	TCCGACTTGTCTGCCAAGAT	101	GQ395696
*pparα*	CTGATAAAGCTTCGGGCTTCCA	CGCTCACACTTATCATACTCCAGCT	106	KF871430
*acly*	AAAAGCTTTGATGAGCTTGGGG	TACAGTGGGAGGAGGCAACTCTT	102	XM_003442027
*accα*	TAGCTGAAGAGGAGGGTGCAAGA	AACCTCTGGATTGGCTTGAACA	110	XM_005471970
*fas*	TCATCCAGCAGTTCACTGGCATT	TGATTAGGTCCACGGCCACA	102	GU433188
*srebp*-1*c*	TGCAGCAGAGAGACTGTATCCGA	ACTGCCCTGAATGTGTTCAGACA	102	XM_005457771
*dgat*2	GCTTGAATTCTGTCACCCTGAAGA	ACCTGCTTGTAGGCGTCGTTCT	106	XM_003458972
*ef*1*a*	ATCAAGAAGATCGGCTACAACCCT	ATCCCTTGAACCAGCTCATCTTGT	109	KJ123689.1
*β-actin*	AGCCTTCCTTCCTTGGTATGGAAT	TGTTGGCGTACAGGTCCTTACG	102	KJ126772.1

*Note. atg*5, autophagy-related 5; *atg*12, autophagy-related 12; *lc*3*a*, microtubule-associated protein 1 light chain 3 alpha; *lc*3*b*, microtubule associated protein 1 light chain 3 beta; *perilipin*2; *atgl*, adipose triglyceride lipase; *hsl*, hormone-sensitive lipase; *mgl*, monoacylglycerol lipase; *aco*, acyl-CoA oxidase; *accβ*, acetyl-CoA carboxylase beta; *cpt*1*a*, carnitine palmitoyltransferase 1a; *cpt*1*b*, carnitine palmitoyltransferase 1b; *pparα*, peroxisome proliferator-activated receptor *α*; *acly*, ATP citrate lyase; *accα*, acetyl-CoA carboxylase *α*; *fas*, fatty acid synthase; *dgat*2, diacylglycerol O-acyltransferase 2; *srebp*-1*c*, sterol regulatory element-binding transcription factor 1c; *ef*1*α*, elongation factor 1 alpha.

**Table 4:** Effects of dietary EA levels on the growth performance of Nile tilapia (*Oreochromis niloticus*).

Indexes	EA0	EA1	EA2	EA3	EA4	EA5
IBW (g)	3.01 ± 0.00	3.00 ± 0.01	3.01 ± 0.00	3.00 ± 0.01	3.00 ± 0.00	3.01 ± 0.00
FBW (g)	35.91 ± 0.68	33.35 ± 0.44	34.70 ± 1.27	35.24 ± 0.38	34.91 ± 0.87	34.69 ± 1.33
WG (%)	1,096.46 ± 20.75	1,013.47 ± 22.81	1,057.36 ± 35.69	1,074.61 ± 13.17	1,061.17 ± 25.68	1,052.32 ± 36.99
SGR (%/day)	4.43 ± 0.08	4.30 ± 0.09	4.37 ± 0.13	4.40 ± 0.05	4.38 ± 0.10	4.36 ± 0.14
HSI (%)	2.27 ± 0.08^a^	2.41 ± 0.03^a^	2.45 ± 0.07^ab^	2.53 ± 0.11^ab^	2.73 ± 0.07^b^	2.57 ± 0.10^ab^
MFI (%)	0.46 ± 0.03^a^	0.58 ± 0.04^a^	0.59 ± 0.06^a^	0.57 ± 0.05^a^	0.83 ± 0.07^b^	0.68 ± 0.06^ab^
FE	0.96 ± 0.00	0.96 ± 0.01	0.96 ± 0.02	0.98 ± 0.00	0.97 ± 0.03	0.98 ± 0.02

*Note*. IBW, initial body weight; FBW, final body weight; WG, weight gain (%) = 100 × (final body weight−initial body weight) / initial body weight; SGR, specific growth rate (%/day) = 100 × (ln (final body weight) − ln (initial body weight)) / days; HSI, hepatosomatic index (%) = 100 × liver weight (g) /fish weight (g); MFI, mesenteric fat index (%) = 100 × mesenteric fat weight (g) / fish weight (g). FE, feed efficiency = fish weight gain / feed intake. EA0, EA1, EA2, EA3, EA4 and EA5: the experimental groups with different contents of EA (0, 3, 6, 12, 20, and 27 g/kg diet). Values are means ± SEM (*n* = 3 (WG, SGR, FE); *n* = 16 (HSI, MFI)), and different superscript letters indicate significant differences (*P*  < 0.05).

**Table 5:** Effects of dietary EA levels on proximate compositions of the whole body, muscle, and liver of tilapia (wet weight (g/kg)).

Parameters	EA0	EA1	EA2	EA3	EA4	EA5
Whole body	142.0 ± 5.5^b^	127.0 ± 2.7^a^	130.1 ± 3.7^ab^	127.4 ± 1.1^a^	127.4 ± 2.0^a^	124.3 ± 1.5^a^
Protein (g/kg)						
Lipid (g/kg)	39.5 ± 1.2^a^	38.4 ± 0.3^a^	42.0 ± 2.6^ab^	42.5 ± 3.3^ab^	47.5 ± 3.3^ab^	50.7 ± 0.8^b^
Moisture (g/kg)	758.2 ± 3.9^ab^	760.0 ± 2.2^b^	758.2 ± 3.1^ab^	756.3 ± 2.9^ab^	754.3 ± 5.1^ab^	743.3 ± 4.2^a^
Ash (g/kg)	22.9 ± 0.1	22.4 ± 0.1	26.5 ± 1.2	27.2 ± 1.6	26.6 ± 2.3	25.7 ± 1.1
Muscle						
Protein (g/kg)	177.7 ± 0.4^c^	174.8 ± 0.2^ab^	173.3 ± 0.5^ab^	174.7 ± 0.1^ab^	175.3 ± 0.2^b^	174.6 ± 0.3^a^
Lipid (g/kg)	15.1 ± 0.4^a^	15.0 ± 0.8^a^	16.6 ± 1.2^ab^	15.6 ± 0.6^ab^	16.9 ± 1.0^ab^	19.0 ± 0.7^b^
Moisture (g/kg)	791.6 ± 1.9	789.1 ± 1.0	791.2 ± 2.6	792.5 ± 1.5	792.3 ± 0.9	789.8 ± 0.2
Ash (g/kg)	12.1 ± 0.2	12.0 ± 0.1	12.3 ± 0.4	11.7 ± 0.0	12.1 ± 0.3	11.7 ± 0.4
Liver						
Lipid (g/kg)	62.9 ± 1.0^a^	74.0 ± 4.6^ab^	81.3 ± 2.9^b^	80.2 ± 3.0^b^	82.2 ± 3.8^b^	103.4 ± 5.6^c^
Moisture (g/kg)	703.0 ± 4.2	706.8 ± 5.7	704.6 ± 2.0	704.8 ± 3.2	699.4 ± 2.7	692.8 ± 3.5

EA0, EA1, EA2, EA3, EA4, and EA5: the experimental groups with different contents of EA (0, 3, 6, 12, 20, and 27 g/kg diet). Values are means ± SEM (*n* = 5) and values in the same row with different superscript letters are significantly different (*P*  < 0.05).

**Table 6:** Effects of dietary EA levels on serum and liver biochemical indicators of tilapia.

Treatments	EA0	EA1	EA2	EA3	EA4	EA5
Serum						
TG (mmol/L)	1.81 ± 0.09^a^	2.3 ± 0.10^c^	1.94 ± 0.09^ab^	2.36 ± 0.09^bc^	3.04 ± 0.14^d^	3.57 ± 0.12^e^
TC (mmol/L)	2.18 ± 0.65^a^	2.05 ± 0.02^a^	2.10 ± 0.05^a^	2.05 ± 0.03^a^	2.68 ± 0.06^b^	2.84 ± 0.04^b^
LDL-C (mmol/L)	0.26 ± 0.01^a^	0.35 ± 0.02^b^	0.38 ± 0.02^b^	0.43 ± 0.02^b^	0.69 ± 0.03^c^	0.76 ± 0.02^c^
HDL-C (mmol/L)	1.41 ± 0.09^b^	1.04 ± 0.03^ab^	1.19 ± 0.04^bc^	1.00 ± 0.04^ab^	0.96 ± 0.04^a^	0.87 ± 0.03^a^
NEFA (mmol/L)	0.20 ± 0.01^ab^	0.20 ± 0.01^ab^	0.16 ± 0.01^a^	0.18 ± 0.02^a^	0.24 ± 0.02^b^	0.24 ± 0.01^b^
GLU (mmol/L)	2.97 ± 0.15^c^	2.52 ± 0.1^b^	2.55 ± 0.10^bc^	2.57 ± 0.07^bc^	2.56 ± 0.12^bc^	1.78 ± 0.08^a^
INS (pmol/L)	46.03 ± 1.92^a^	52.22 ± 1.8^ab^	58.74 ± 4.38^abc^	60.68 ± 4.97^abc^	67.55 ± 2.6^c^	64.88 ± 3.53^bc^
MDA (nmol/L)	5.17 ± 0.18^ab^	4.92 ± 0.28^ab^	4.79 ± 0.2^ab^	4.65 ± 0.35^a^	5.10 ± 0.23^ab^	5.98 ± 0.36^b^
H_2_O_2_ (mmol/L)	47.73 ± 1.61^b^	45.78 ± 2.15^ab^	41.07 ± 1.74^ab^	38.31 ± 1.01^a^	48.38 ± 1.42^b^	46.76 ± 2.65^b^
AST (U/L)	3.84 ± 0.39^a^	9.75 ± 0.98^b^	9.50 ± 1.34^b^	10.2 ± 0.84^b^	12.6 ± 1.08^bc^	14.93 ± 1.05^c^
ALT (U/L)	7.49 ± 0.51^a^	10.00 ± 0.94^ab^	11.95 ± 1.08^bc^	11.08 ± 0.39^b^	12.79 ± 0.89^bc^	15.07 ± 0.75^c^
T-AOC (mmol/L)	0.41 ± 0.01^c^	0.37 ± 0.00^b^	0.33 ± 0.01^a^	0.33 ± 0.01^a^	0.33 ± 0.01^a^	0.33 ± 0.01^a^
T-SOD (U/mL)	47.09 ± 2.05^d^	36.53 ± 1.04^bc^	40.47 ± 1.5^cd^	29.66 ± 2.01^ab^	25.47 ± 3.56^a^	23.24 ± 1.53^a^
CAT (U/mL)	6.71 ± 0.35^c^	6.96 ± 0.75^c^	5.79 ± 0.27^bc^	4.47 ± 0.28^ab^	2.55 ± 0.22^a^	4.48 ± 0.27^ab^
Liver						
TG (mmol/gprot)	0.57 ± 0.04^a^	0.48 ± 0.03^a^	0.59 ± 0.03^ab^	0.69 ± 0.08^ab^	0.79 ± 0.05^b^	0.8 ± 0.07^b^
TC (mmol/gprot)	0.067 ± 0.003^ab^	0.065 ± 0.002^a^	0.070 ± 0.002^ab^	0.066 ± 0.003^ab^	0.074 ± 0.002^b^	0.069 ± 0.002^ab^
NEFA (mmol/gprot)	0.20 ± 0.01^ab^	0.18 ± 0.01^a^	0.21 ± 0.01^ab^	0.22 ± 0.01^ab^	0.24 ± 0.01^ab^	0.27 ± 0.02^b^
MDA (nmol/gprot)	0.99 ± 0.04^a^	1.10 ± 0.00^abc^	1.09 ± 0.04^ab^	1.06 ± 0.02^ab^	1.29 ± 0.09^b^	1.25 ± 0.05^bc^
H_2_O_2_ (mmol/gprot)	2.62 ± 0.18^a^	3.29 ± 0.25^a^	3.35 ± 0.27^a^	4.11 ± 0.34^ab^	4.21 ± 0.35^ab^	5.49 ± 0.65^b^
T-AOC (mmol/gprot)	0.07 ± 0.003^b^	0.07 ± 0.002^b^	0.07 ± 0.002^b^	0.07 ± 0.002^b^	0.07 ± 0.002^b^	0.06 ± 0.001^a^
T-SOD (U/mgprot)	1.57 ± 0.05^b^	1.71 ± 0.06^b^	1.69 ± 0.08^b^	1.57 ± 0.04^b^	1.51 ± 0.03^ab^	1.30 ± 0.05^a^
CAT (U/mgprot)	95.19 ± 4.54^b^	85.11 ± 2.03^a^	88.39 ± 0.99^ab^	85.51 ± 2.29^a^	85.53 ± 2.36^a^	84.56 ± 1.93^a^

*Notes*. TG, triglyceride; TC, total cholesterol; LDL-C, low-density lipoprotein cholesterol; HDL-C, high-density lipoprotein cholesterol; NEFA, nonesterified fatty acids; GLU, glucose; INS, insulin; MDA, malondialdehyde; H_2_O_2_, hydrogen peroxide; AST, aspartate aminotransferase; ALT, alanine aminotransferase; T-AOC, total antioxidant capacity; T-SOD, total superoxide dismutase; CAT, catalase. EA0, EA1, EA2, EA3, EA4, and EA5: the experimental groups with different contents of EA (0, 3, 6, 12, 20, and 27 g/kg diet). Values are presented as means ± SEM (*n* = 8), and different superscripts letters indicate significant differences (*P*  < 0.05).

**Table 7:** Fatty acid composition of total lipids (total fatty acids (g/kg)) in the liver of tilapia.

Fatty acids	EA0	EA1	EA2	EA3	EA4	EA5
C14 : 0	37.8 ± 1.7^ab^	35.2 ± 1.7^a^	37.1 ± 1.9^ab^	43.6 ± 1.8^ab^	45.5 ± 3.6^ab^	50.5 ± 5.6^b^
C16 : 0	341.6 ± 11.7^bc^	377.0 ± 7.8^c^	359.7 ± 6.0^bc^	338.2 ± 11.8^abc^	335.2 ± 5.9^ab^	297.5 ± 10.7^a^
C16 : 1n-7	62.9 ± 2.0^a^	79.4 ± 3.8^b^	77.3 ± 3.4^ab^	78.3 ± 5.4^b^	74.6 ± 1.4^ab^	70.1 ± 3.1^ab^
C18 : 0	104.9 ± 2.2^c^	100.6 ± 4.8^bc^	101.5 ± 3.6^bc^	88.4 ± 1.7^ab^	93.3 ± 0.6^bc^	76.7 ± 3.4^a^
C18 : 1n-9	323.2 ± 12.8^b^	276.2 ± 7.7^a^	291.4 ± 4.9^ab^	285.7 ± 12.3^ab^	273.6 ± 5.1^a^	278.2 ± 5.4^a^
C18 : 2n-6	43.5 ± 2.0^ab^	39.9 ± 3.8^a^	37.3 ± 1.3^a^	45.2 ± 4.9^ab^	43.7 ± 1.6^ab^	57.3 ± 6.2^b^
C18 : 3n-3	3.9 ± 0.3^ab^	4.0 ± 0.6^ab^	3.0 ± 0.1^a^	4.0 ± 0.5^ab^	3.7 ± 0.1^ab^	5.5 ± 1.0^b^
C18 : 3n-6	6.9 ± 0.3^ab^	7.3 ± 0.3^ab^	6.2 ± 0.4^a^	10.1 ± 1.6^bc^	8.4 ± 0.3^ab^	12.4 ± 1.0^c^
C20 : 1n-9	13.6 ± 1.0^ab^	11.2 ± 0.9^a^	13.6 ± 0.3^ab^	15.7 ± 1.0^b^	16.6 ± 0.4^bc^	19.9 ± 1.0^c^
C20 : 3n-6	10.7 ± 0.5^a^	9.7 ± 0.3^a^	10.3 ± 1.3^a^	13.2 ± 1.4^a^	13.7 ± 0.8^a^	17.8 ± 0.7^b^
C20 : 4n-6	28.7 ± 1.0	30.7 ± 1.6	29.3 ± 2.8	31.3 ± 2.9	35.7 ± 1.5	36.9 ± 1.5
C20 : 5n-3	1.1 ± 0.1^a^	1.1 ± 0.1^a^	1.2 ± 0.1^a^	2.5 ± 0.1^b^	2.3 ± 0.1^b^	2.6 ± 0.2^b^
C22 : 0	6.1 ± 0.6	7.6 ± 0.9	7.5 ± 0.8	6.6 ± 0.5	6.4 ± 0.3	5.7 ± 0.3
C22 : 1n-9	nd	4.4 ± 0.5^ab^	9.4 ± 0.9^b^	20.6 ± 1.5^c^	28.6 ± 2.2^c^	48.7 ± 4.0^d^
C22 : 6n-3	15.1 ± 0.6	15.6 ± 0.7	15.1 ± 1.8	16.6 ± 1.9	18.9 ± 0.4	20.1 ± 1.0
Total SFA	490.5 ± 12.4^bc^	520.4 ± 4.9^c^	505.8 ± 1.9^bc^	476.7 ± 12.8^b^	480.4 ± 8.2^bc^	430.4 ± 10.3^a^
Total MUFA	399.8 ± 12.0^ab^	371.2 ± 4.5^a^	391.7 ± 5.3^ab^	400.3 ± 13.0^ab^	393.3 ± 6.7^ab^	417.0 ± 8.1^b^
Total PUFA	109.8 ± 2.1^a^	108.4 ± 2.0^a^	102.5 ± 6.4^a^	123.0 ± 10.0^a^	126.3 ± 2.1^ab^	152.7 ± 9.3^b^
Total n-3 PUFA	20.0 ± 0.6^a^	20.7 ± 0.4^a^	19.4 ± 1.9^a^	23.1 ± 2.2^ab^	24.9 ± 0.4^ab^	28.3 ± 1.7^b^
Total n-6 PUFA	89.7 ± 1.9^a^	87.7 ± 2.2^a^	83.1 ± 4.6^a^	99.9 ± 8.2^a^	101.4 ± 2.0^a^	124.4 ± 7.8^b^
n-3/n-6	0.22 ± 0.01	0.24 ± 0.01	0.23 ± 0.01	0.23 ± 0.01	0.25 ± 0.01	0.23 ± 0.01

*Note*. MUFA, monounsaturated fatty acids; PUFA,polyunsaturated fatty acids; SFA, saturated fatty acids. Total SFA includes 14 : 0, 16 : 0, 18 : 0, and 22 : 0; total MUFA includes 16 : 1n-7, 18 : 1n-9, 20 : 1n-9, and 22 : 1n-9; total PUFA includes 18 : 2n-6, 18 : 3n-3, 18 : 3n-6, 20 : 3n-6, 20 : 4n-6, 20 : 5n-3, and 22 : 6n-3; total n-3 PUFA includes 18 : 3n-3, 20 : 5n-3, and 22 : 6n-3; total n-6 PUFA includes 18 : 2n-6, 18 : 3n-6, 20 : 3n-6, and 20 : 4n-6. nd, not detected; EA0, EA1, EA2, EA3, EA4 and EA5: the experimental groups with different contents of EA (0, 3, 6, 12, 20, and 27 g/kg diet). Values are means ± SEM (*n* = 5) and values in the same row with different superscript letters are significantly different (*P* < 0.05).

**Table 8:** Fatty acid composition of total lipids (total fatty acids (g/kg)) in the muscle of tilapia.

Fatty acids	EA0	EA1	EA2	EA3	EA4	EA5
C14 : 0	21.7 ± 0.8^a^	20.0 ± 1.3^a^	24.7 ± 1.1^ab^	24.6 ± 1.2^ab^	25.4 ± 2.8^ab^	29.6 ± 1.3^b^
C16 : 0	423.7 ± 3.3^d^	378.6 ± 8.4^c^	376.6 ± 4.1^c^	354.9 ± 9.8^c^	300.5 ± 7.2^b^s	264.0 ± 9.1^a^
C16 : 1n-7	11.9 ± 0.4	11.7 ± 0.5	12.1 ± 0.3	12.3 ± 0.4	11.4 ± 0.4	12.6 ± 0.7
C18 : 0	97.9 ± 2.0^d^	81.4 ± 3.5^c^	73.4 ± 2.8^bc^	76.7 ± 3.3^bc^	66.3 ± 2.4^ab^	57.6 ± 2.3^a^
C18 : 1n-9	233.0 ± 8.0^a^	222.8 ± 7.0^a^	245.1 ± 6.5^ab^	250.4 ± 8.3^ab^	266.8 ± 5.5^b^	272.4 ± 7.5^b^
C18 : 2n-6	106.5 ± 4.7^a^	134.4 ± 3.2^b^	126.0 ± 1.8^b^	127.9 ± 5.9^b^	128.9 ± 1.1^b^	130.9 ± 4.7^b^
C18 : 3n-3	8.5 ± 0.7^a^	14.6 ± 0.5^b^	14.8 ± 0.5^b^	13.4 ± 1.2^b^	15.6 ± 0.6^b^	16.3 ± 0.4^b^
C18 : 3n-6	5.9 ± 0.2^a^	12.5 ± 0.6^b^	11.1 ± 0.7^b^	11.8 ± 1.3^b^	13.6 ± 0.8^bc^	16.8 ± 0.3^c^
C20 : 1n-9	12.9 ± 0.9^a^	12.2 ± 0.8^a^	14.4 ± 0.4^a^	18.0 ± 0.7^b^	23.0 ± 0.8^c^	24.3 ± 0.8^c^
C20 : 3n-6	18.4 ± 1.1	17.4 ± 1.1	13.0 ± 1.9	16.0 ± 1.2	17.2 ± 1.5	16.9 ± 0.8
C22 : 0	5.6 ± 0.3^a^	8.7 ± 2.5^ab^	8.7 ± 1.4^ab^	7.1 ± 0.9^ab^	11.1 ± 0.7^b^	5.7 ± 0.2^ab^
C20 : 4n-6	27.8 ± 6.4^a^	47.6 ± 3.0^b^	38.4 ± 3.0^ab^	38.4 ± 5.0^ab^	42.4 ± 3.0^ab^	37.4 ± 2.1^ab^
C20 : 5n-3	2.8 ± 0.3^a^	4.4 ± 0.2^c^	3.9 ± 0.2^bc^	4.0 ± 0.4^bc^	3.6 ± 0.2^abc^	3.0 ± 0.1^ab^
C22 : 1n-9	nd	6.2 ± 0.4^ab^	15.7 ± 1.6^bc^	26.9 ± 2.5^c^	49.2 ± 6.5^d^	62.9 ± 4.4^d^
C22 : 6n-3	23.7 ± 1.0^ab^	27.4 ± 2.3^b^	22.2 ± 2.5^ab^	17.6 ± 3.0^a^	24.8 ± 2.1^ab^	22.2 ± 1.4^ab^
Total SFA	548.8 ± 4.1^d^	488.7 ± 5.9^c^	483.4 ± 5.1^c^	463.3 ± 12.6^c^	403.3 ± 9.0^b^	356.9 ± 12.2^a^
Total MUFA	257.7 ± 9.1^a^	252.9 ± 7.7^a^	287.3 ± 8.2^ab^	307.6 ± 9.4^b^	350.5 ± 11.3^c^	372.2 ± 11.1^c^
Total PUFA	193.4 ± 11.6^a^	258.4 ± 7.6^b^	229.3 ± 8.8^ab^	229.1 ± 15.7^ab^	246.2 ± 5.5^b^	243.5 ± 9.3^b^
Total n-3 PUFA	35.0 ± 1.8^a^	46.4 ± 2.2^b^	40.9 ± 2.7^ab^	35.0 ± 4.0^a^	44.0 ± 1.6^ab^	41.4 ± 1.9^ab^
Total n-6PUFA	158.5 ± 10.0^a^	212.0 ± 5.5^b^	188.5 ± 6.3^ab^	194.1 ± 12.4^b^	202.2 ± 4.1^b^	202.1 ± 7.5^b^
n-3 /n-6	0.22 ± 0.01^b^	0.22 ± 0.01^b^	0.22 ± 0.01^b^	0.18 ± 0.01^a^	0.22 ± 0.01^b^	0.20 ± 0.01^ab^

*Note*. MUFA, monounsaturated fatty acids; PUFA, polyunsaturated fatty acids; SFA, saturated fatty acids. Total SFA includes 14 : 0, 16 : 0, 18 : 0, and 22 : 0; total MUFA includes 16 : 1n-7, 18 : 1n-9, 20 : 1n-9, and 22 : 1n-9; total PUFA includes 18 : 2n-6, 18 : 3n-3, 18 : 3n-6, 20 : 3n-6, 20 : 4n-6, 20 : 5n-3, and 22 : 6n-3; total n-3 PUFA includes 18 : 3n-3, 20 : 5n-3, and 22 : 6n-3; total n-6 PUFA includes 18 : 2n-6, 18 : 3n-6, 20 : 3n-6, and 20 : 4n-6. nd, not detected; EA0, EA1, EA2, EA3, EA4, and EA5: the experimental groups with different contents of EA (0, 3, 6, 12, 20, and 27 g/kg diet). Values are means ± SEM (*n* = 5), and values in the same row with different superscript letters are significantly different (*P*  < 0.05).

## Data Availability

The data used to support the findings of this study are available from the corresponding author upon request.
